# Transference Numbers and Ion Coordination Strength for Mg^2+^, Na^+^, and K^+^ in Solid Polymer Electrolytes

**DOI:** 10.1021/acs.jpcc.4c04632

**Published:** 2024-09-23

**Authors:** Rassmus Andersson, Caroline Mönich, Guiomar Hernández, Monika Schönhoff, Jonas Mindemark

**Affiliations:** † Department of Chemistry, Ångström Laboratory, Uppsala University, Box 538, SE-751 21 Uppsala, Sweden; ‡ Institute of Physical Chemistry, University of Münster, Corrensstraße 28/30, 48149 Münster, Germany

## Abstract

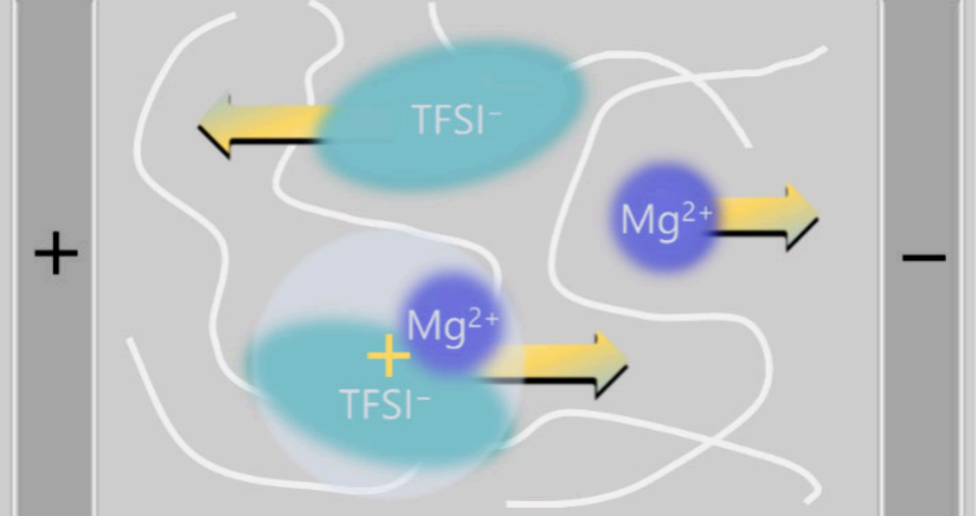

In solid polymer electrolytes, the transference number (*T*_*i*_) is fundamental for performance as it defines the transport efficiency of the electrochemically active species. For systems “beyond Li”, a lack of suitable methods to determine the *T*_*i*_ is limiting the understanding of the ion transport in such systems. In this study, a method based on electrophoretic NMR (eNMR) and electrochemical impedance spectroscopy (EIS) is used to determine the *T*_+_ for Na^+^, K^+^, and Mg^2+^ in poly(ε-caprolactone) (PCL), comparing this to previous results in poly(ethylene oxide). In common for all cations, a distinct correlation between strong coordination and a low *T*_+_ is observed. Paradoxically, however, the divalent Mg^2+^ in PCL displayed a *T*_+_ of 0.86 compared to ∼0.5 for the monovalent cations. Persistent clustering in this system suggests that it is better treated as a monovalent [MgTFSI]^+^ + TFSI^–^ system, resulting in a *T*_+_ of the [MgTFSI]^+^ cation of 0.43. These results serve as a door opener for the wide applicability of the eNMR/EIS method in order to provide an understanding of charge transport in multivalent systems.

## Introduction

The rapidly increasing demand for energy storage devices has turned the attention toward alternative technologies based on more abundant resources than Li for “next-generation” batteries. The neighbors of Li in the periodic table – Na, K and Mg, having an almost thousandfold higher terrestrial abundance–are of prime interest and are central to the currently emerging “beyond Li” technologies.^1^ Solid polymer electrolytes (SPEs) are another emerging technology for “next-generation” batteries, due to their compatibility with the metallic form of the cation in the system as the negative electrode, which intrinsically has a higher specific capacity compared to the carbon-based electrodes used in metal-ion batteries today.^2^ The SPEs also have the advantage of being less volatile than the liquid electrolytes typically used, which makes them less flammable.^3^ A combination of these “beyond Li” technologies with SPEs is believed to be a possible way forward to cover the demand for safe and sustainable stationary energy storage. While a lot is known about Li^+^ conduction in SPEs, the behavior of cations beyond Li is still unclear. One of the main parameters used to investigate ion conduction is the transference number (*T*_*i*_), which can be defined as the effective molar transport by electric field-driven migration of a certain species per Faraday of charge passed. In a battery where only the cation is electrochemically active, the cation transference number *T*_+_ ideally is equal to 1, meaning that all the current is derived from cation transport with no contribution from the anions. In practice, most salt-in-polymer electrolytes remain distinctly below this value. For example, for salt-in-poly(ethylene oxide), typical values are on the order of 0.2.^4,5^ A higher *T*_+_ is favorable since it prevents the formation of large concentration gradients in the SPE, as such gradients reduce the performance of the cell and can potentially even lead to cell failure due to the depletion of cations in the vicinity of the negative electrode.

Several approaches can be used to determine the *T*_+_ for Li; however, many rely on conditions and assumptions that cannot always be met or combinations of methods, resulting in larger errors for the derived *T*_+_.^6−11^ Furthermore, when stating *T*_*+*_ values, it is important to mention the reference frame in which they are determined. Some confusion arose in the literature since *T*_*+*_ values determined in the solvent-fixed frame do not necessarily match those determined by other methods when the solvent is drifting.^4,5,12^ A reconciliation is provided by suitable reference frame transformations.^13,14^ Moving forward to cation systems beyond Li, the challenges of metal electrodes for *T*_+_ determination intensify, due to intrinsically more reactive metals and difficulties in achieving a stable potential and interfacial resistance.^15−17^

For Li, *T*_+_ can also be determined with NMR-based methods. An established procedure involves the method of Pulsed Field Gradient (PFG)-NMR, from which diffusion coefficients are obtained. This yields the statistical Brownian motion of species, no matter whether they are anionic, cationic, or neutral. With suitable assumptions, involving the Nernst–Einstein equation, information about the drift of ionic species in an electric field can be derived.^8^ Although this approach is widely employed in electrolyte research, it lacks precision, as it completely neglects ion correlations, which are ubiquitous in concentrated electrolytes. Therefore, more recently electrophoretic NMR (eNMR) was introduced to concentrated electrolytes.^18,19^ It allows the direct determination of the ionic drift velocities in an electric field and the subsequent calculation of transference numbers. In this way, *T*_+_ values were obtained for a range of Li^+^-conducting polymer electrolytes.^20^ Concerning the reference frame of the eNMR measurements, it was previously shown that mobilities are measured in a volume-fixed reference frame, and transference numbers can easily be converted to other frames of reference.^21,22^ However, for the “next-generation” cations, a major obstacle for NMR-based methods is the very low natural abundance of NMR-active isotopes and/or too short spin relaxation times, which prevent NMR transport measurements.

This lack of methods to determine *T*_+_ has effectively hindered the investigation of ion transport-correlating factors beyond Li. The ion coordination strength, for example is a parameter that has been found to correlate strongly with *T*_+_, where strong ion coordination gives a low *T*_+_ and vice versa.^20^ While this correlation has so far only been demonstrated for Li-based systems due to the aforementioned lack of reliable methods to determine the *T*_+_ for other cations, it has nevertheless been shown that similar coordination strength trends are seen for Li, Na and Mg with different polymer systems.^23^

Recently, to address the challenge of post-Li transference number measurements, eNMR has been combined with electrochemical impedance spectroscopy (EIS) to determine the *T*_+_ for Na, K and Mg in poly(ethylene oxide) (PEO).^24^ In short, the method relies on the total ionic conductivity (σ_tot_), which is the sum of all partial conductivities, and in the case of one cation and one anion, it equals the sum of σ_+_ and σ_–_. Then eNMR is used to determine σ_–_ and EIS to determine σ_tot_, from which *T*_+_ can be calculated. This method, termed eNMR/EIS, opens a route to quantify *T*_+_ for any type of cation, regardless of the number of ionic species in the system, as long as the number of constituents not detectable by NMR is limited to one.^24^ Furthermore, it provides an opportunity to examine the correlation between *T*_+_ and the ion coordination strength–as already observed for Li–for any type of cation and SPE. We note that a similar combination of EIS and eNMR has also been used to obtain the mobility of an undetectable anion in an ionic liquid.^25^ In this work, we determine *T*_*+*_ for a series of salts in poly(ε-caprolactone) (PCL) with different cations using eNMR/EIS. The similarities of PEO and PCL in terms of chain dynamics (as measured by the glass transition temperatures) makes it relatively straightforward to correlate the results of a comparison between these two materials to the coordination strength for the respective cation.

## Experimental Section

### Materials and Sample Preparation

Magnesium(II) bis(trifluoromethanesulfonyl)imide (Mg(TFSI)_2_), sodium bis(trifluoromethanesulfonyl)imide (NaTFSI) and potassium bis(trifluoromethanesulfonyl)imide (KTFSI), all with a purity of 99.5% and packed under argon, were purchased from Solvionic. Poly(ε-caprolactone) (PCL) with *M*_n_ = 4000 g mol^–1^ was obtained from Perstorp, Sweden (Capa 2402) and poly(ethylene oxide) (PEO) with *M*_n_ = 4000 g mol^–1^ was purchased from Sigma-Aldrich. The polymer electrolytes were prepared by solution casting of dissolved salts and polymers in acetonitrile and evaporated under controlled conditions as described elsewhere.^20^ The salt concentration was kept at a ratio of *r* = 0.1 (metal ion/monomer) for the NaTFSI and KTFSI samples, whereas the Mg(TFSI)_2_ samples were prepared with *r* = 0.05 in order to keep a comparable anion concentration.

### Fourier Transform Infrared Spectroscopy (FTIR)

The ion coordination strength measurements were conducted in an ATR-FTIR setup with a VERTEX 70v FTIR Spectrometer from Bruker with an RT-DLaTGS detector and a Quest ATR Accessory with a Diamond heated puck from Specac. The measurements were performed in the temperature range 25–100 °C at intervals of 15 °C with 32 scans and a resolution of 4 cm^–1^.

### Pulsed Field Gradient (PFG)-NMR Diffusion

The measurements were performed on an Avance III HD spectrometer with a static field of 9.39 T using a DiffBBO probe head (Bruker, Rheinstetten), applying a stimulated echo sequence. The sample temperature was 90 °C. ^19^F-based diffusion experiments were performed with an observation time of Δ = 0.1 s, gradient pulses of length δ = 1 ms (K- and Na- containing samples) or 2 ms (Mg-containing samples) and a maximum gradient strength *g*_max_ between 6.2 T m^–1^ and 11 T m^–1^. The anion diffusion coefficients were obtained by an exponential fit of the echo decay.

### Electrophoretic Nuclear Magnetic Resonance Spectroscopy (eNMR)

Employing the same spectrometer, a double stimulated echo sequence was used, including two voltage pulses of opposite sign. The latter were applied by a pulse generator, employing a sample holder with electrodes according to a custom design published earlier.^19^ 60 capillaries (Molex-Polymicro Technologies) were placed between the electrodes to suppress convection. The gradient strength *g* was set between 3.85–4.5 T m^–1^, depending on the sample. The gradient pulse duration was δ = 1 ms for the Na and K samples and 2 ms for the Mg samples. The observation time Δ was 0.1 s and the sample temperature was 90 °C. In a series of ^19^F spectra, the voltage *U* was applied in incremented steps of 10 V with alternating polarity up to a maximum of 100 V. For charged species migrating in the electric field, the phase shift (φ – φ_0_) of the spectral line is proportional to the drift velocity *v* in the electric field.
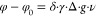
1The phase angle was determined by fits of phase-sensitive Lorentz profiles as described previously.^26^ The electrophoretic mobility μ = *v*/*E* with the electric field strength *E* was then derived from a linear regression of φ – φ_0_ as a function of *E*. It is *E* = *U*/*d*, where *d* is the distance between the electrodes. Mobility measurements were repeated at least three times for each sample, and the results were averaged. Errors resulted from the statistical error (standard deviation), and an additional 5% was estimated for further error sources.

### Electrochemical Impedance Spectroscopy (EIS)

The measurements were performed with a BioLogic SP240 potentiostat in an FN 032 oven from Nüve at 90 °C. The samples were assembled in Swagelok cells with blocking stainless steel electrodes with a fixed diameter of 4 mm. The thickness of the samples was controlled by utilizing 100 μm thick polytetrafluoroethylene ring spacers. Prior to the measurements, the samples were annealed at the measurement temperature for at least 5 h (NaTFSI, KTFSI) and at least 20 h (Mg(TFSI)_2_) to ensure the set measurement temperature was achieved in the cell.

### Density

The density of the samples was determined with an AccuPyc II 1340 gas pycnometer from Micromeritics with helium as the displacement gas. The measurements were performed at 90 °C, the same temperature as the eNMR and EIS measurements.

### Raman Spectroscopy

Raman spectra were collected on samples in 5 mm NMR tubes, using an FT-Raman spectrometer (Bruker, Rheinstetten) equipped with a Nd:YAG Laser (1064 nm) and a power of 263 mW. The resolution was set to 0.5 cm^–1^ with a spectral range of 30–3600 cm^–1^. A baseline correction of the spectra and the subsequent deconvolution were carried out with the OPUS software (Bruker, Rheinstetten).

## Results and Discussion

### Ion Coordination and Interactions

**Figure 1:**
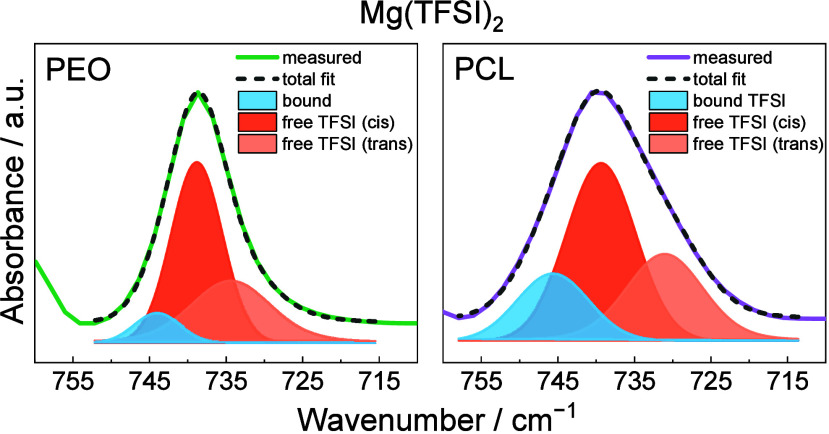
Deconvolution examples of the S–N–S vibration at 740 cm^–1^ of the TFSI anion in FTIR spectra for Mg(TFSI)_2_ in PEO (left) and PCL (right). These representative examples for each sample are taken from triplicates at 85 °C.

In a system merely containing a dissolved salt in a polymer, the dissociation energy (Δ*G*^0^) for the salt dissociation equilibrium is an indirect quantitative measure of the coordination strength between the polymer and the cations, since the salt dissociation is driven by the coordinating ligands in the polymer. For monovalent cation salts, the ion dissociation equilibrium is defined as
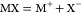
2whereas for divalent cation salts, considering complete dissociation, the equilibrium becomes
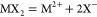
3For the dissociation of a generic monovalent cation (and anion) in a binary salt MX where [M^+^] = [X^–^], the equilibrium constant (eq 2) can be described in terms of the free anion and paired anion concentrations:
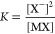
4For divalent cations (and monovalent anions) as in eq 3, the equation changes, since the concentration of the anion is twice that of the cation:
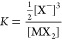
5The absolute concentration of the free and bound TFSI was calculated from the ratios of free and bound TFSI, as determined by deconvolution of FTIR spectra (see example for Mg(TFSI)_2_ in Figure 1 and all cations in Figure S1), and the determined densities of the samples (Table S1). A more elaborate explanation is found in an earlier publication.^23^ Important to note is that the reference point for the salt dissociation is the ion pair itself, meaning that while comparisons of the Δ*G*^0^ between different polymers for a specific salt are valid, any comparisons between different salts should be done with caution.

**Figure 2:**
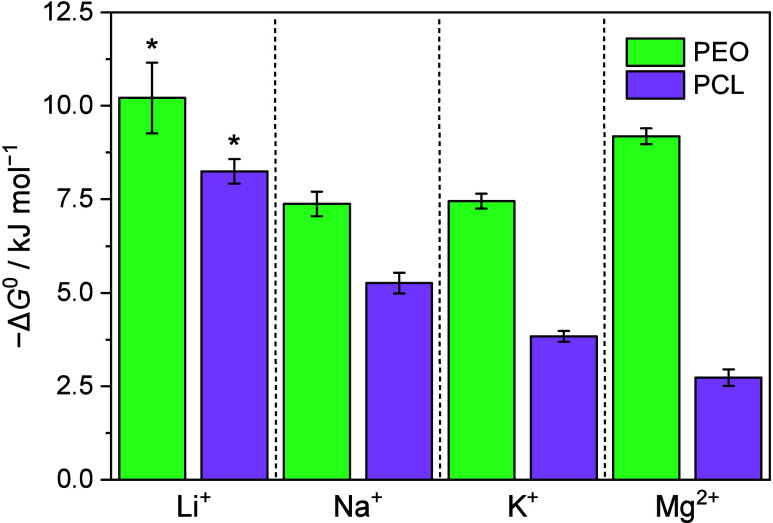
Calculated dissociation energies (Δ*G*^0^) for Li^+^, Na^+^, K^+^, and Mg^2+^ in PEO and PCL at 25 °C. Li data marked with * is taken from ref (23).

As seen in Figure 2, Δ*G*^0^ calculated by extracting Δ*H*^0^ and Δ*S*^0^ from the van’t Hoff plot (Figure S2) demonstrates the contrast between the strongly coordinating PEO and the more weakly coordinating PCL. This agrees with previous studies, where stronger cation coordination was observed in PEO, stemming from chelation effects and the flexibility of the backbone, which provides greater freedom for more optimal coordination environments.^20,27^ The deconvolution seen in Figure 1 and S2, column 1 of PEO is relatively straightforward since the polymer itself does not have any vibrational peaks in the range of the S–N–S vibration in TFSI at 740 cm^–1^. For PCL, on the contrary, it is more challenging as PCL has a broad peak vibration, assigned to the C–H bending mode, in the range of the S–N–S vibration at 740 cm^–1^. Therefore, two different deconvolutions of the S–N–S vibration in TFSI have been performed, where the PCL results in purple exclude the peak vibration from PCL in the deconvolution (Figure 1b and S2, column 2). In contrast, the PCL results in red include the peak (Figure S1, column 3). While the results obtained when including the C–H peak can be argued to be more correct, the results in Figure S3 show that the difference is marginal between the two deconvolutions, i.e. it seems like the C–H peak is contributing evenly to all deconvoluted peak areas of the S–N–S vibration. This indicates that the C–H peak in PCL can be neglected when deconvoluting for simplicity, with sufficiently accurate results, which indeed was also done previously with Li.^23^ Concerning the equilibrium for Mg, it was assumed that Mg(TFSI)_2_ dissociates completely into free Mg^2+^ and TFSI anions and eq 5 was utilized to calculate the equilibrium constant. This assumption is made to be able to separate the S–N–S vibrations of bound and free TFSI in the FTIR deconvolution, as it is not possible to distinguish between TFSI bound as [MgTFSI]^+^ or Mg(TFSI)_2_. Therefore, an alternative analysis of an equilibrium where Mg(TFSI)_2_ is partly dissociated into [MgTFSI]^+^ and TFSI anions cannot be performed based on the FTIR data.

### Ion Transport

**Figure 3:**
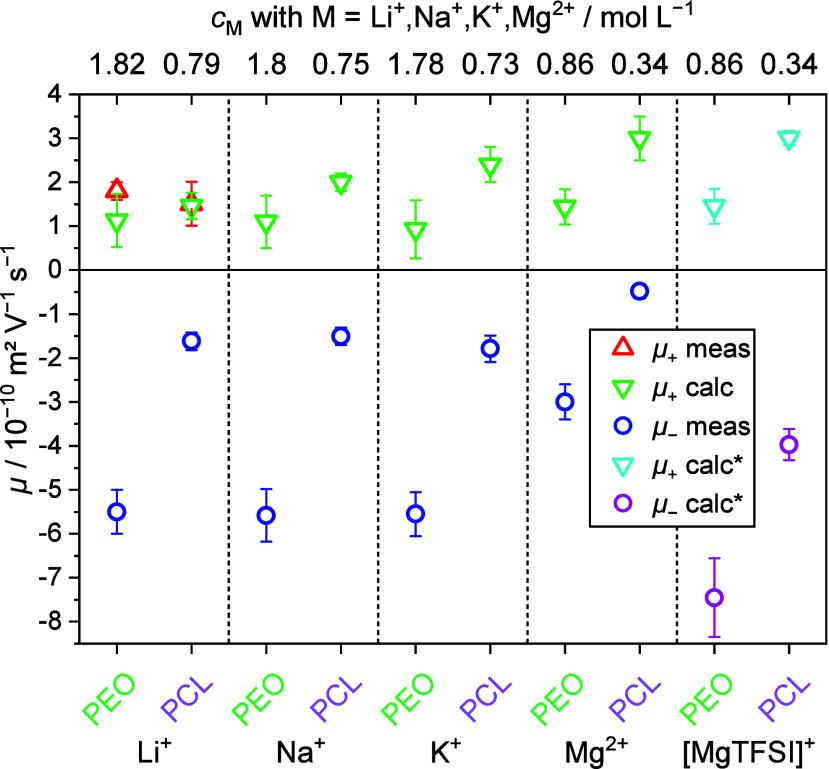
Mobilities for anion (μ_–_, blue symbols, via ^19^F eNMR) and cation (μ_+_, green symbols, calculated via the eNMR/EIS method). The mobilities for the Li systems are reused from ref (20) and mobilities for Na, K, and Mg in PEO are reused from ref (24). Red data points represent directly measured mobilities via ^7^Li eNMR. Data marked with * are values obtained when the Mg system is treated as a monovalent system. For details of this treatment, see eqs 10–13 and the associated discussion.

The addition of PCL – with a clear contrast in ion coordination strength compared to PEO – to the experimental matrix makes it possible to correlate the ion transport of the different cations to the polymer properties, something that has not previously been possible for beyond-Li systems. For systems containing ions with NMR-active isotopes of both the cation and anion, the *T*_+_ determination is relatively straightforward, since the electrophoretic mobilities of both species can be determined, from which the *T*_+_ is calculated. However, for Na, K and Mg, which are investigated in this study, a direct determination of the electrophoretic mobility μ_+_ is not feasible due to low abundance and/or short spin relaxation time of their respective NMR-active isotopes. To circumvent this challenge, the eNMR/EIS method was introduced and applied to determine the *T*_+_ in beyond Li electrolyte systems.^24^ The method is based on the fact that the total conductivity (σ_tot_) in an electrolyte is the result of the total motions of all charge carriers, i.e., cations and anions in a system, where each ionic species *i* contributes with a partial conductivity (σ_*i*_). If the system only contains one cation and one anion species, σ_tot_ is described by the sum of the partial conductivities σ_+_ and σ_–_.
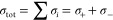
6In the eNMR/EIS method, σ_tot_ is determined by EIS measurements and the respective σ_–_ of the anion species is determined by directly measuring its electrophoretic mobility μ_–_ via ^19^F eNMR, from which σ_–_ is calculated:
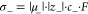
7where *F* is Faraday’s constant, *z* is the charge number of the cation or anion, and *c* is the concentration of the cation or anion. The cation conductivity σ_+_ can be calculated analogously. By rearranging eq 6, σ_+_ can be calculated from σ_tot_ and σ_–_:
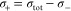
8From the determined conductivities, *T*_+_ is finally calculated:
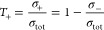
9While this method was previously applied to PEO- and glyme-based electrolytes,^24^ we use it here to study the PCL-based polymer electrolytes with different cations. The respective anion and cation mobilities for TFSI salts are given in Figure 3, alongside with mobilities of the same ions in PEO. In order to apply eq 7, the concentrations are calculated from the densities (Table S1), yielding σ_–_.

**Figure 4:**
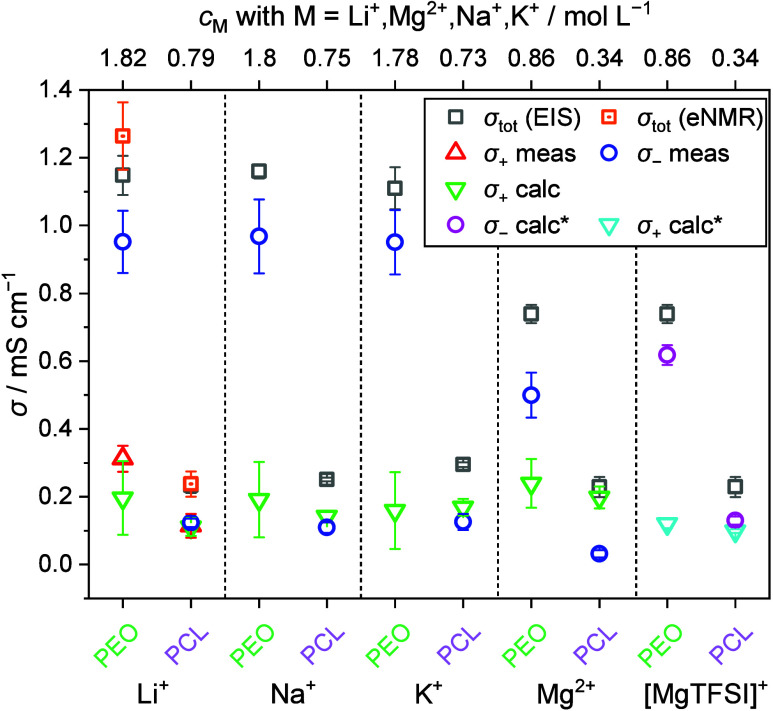
σ_tot_ determined by EIS, σ_tot_ calculated from eNMR measurements (only for the Li systems), σ_*–*_ calculated from eNMR, and σ_*+*_ calculated by the eNMR/EIS approach for LiTFSI, NaTFSI, KTFSI with *r* = 0.1 and Mg(TFSI)_2_ with *r* = 0.05 in PCL (4000 g mol^–1^) at 90 °C. Calculated σ_*+*_ and σ_*–*_ marked with * are values obtained when the Mg system is treated as a monovalent system. For details of this treatment, see eq 10–13 and the associated discussion. The data for the Li systems are reused from ref (20) and for the Na, K, and Mg systems with PEO are reused from ref (24).

**Figure 5:**
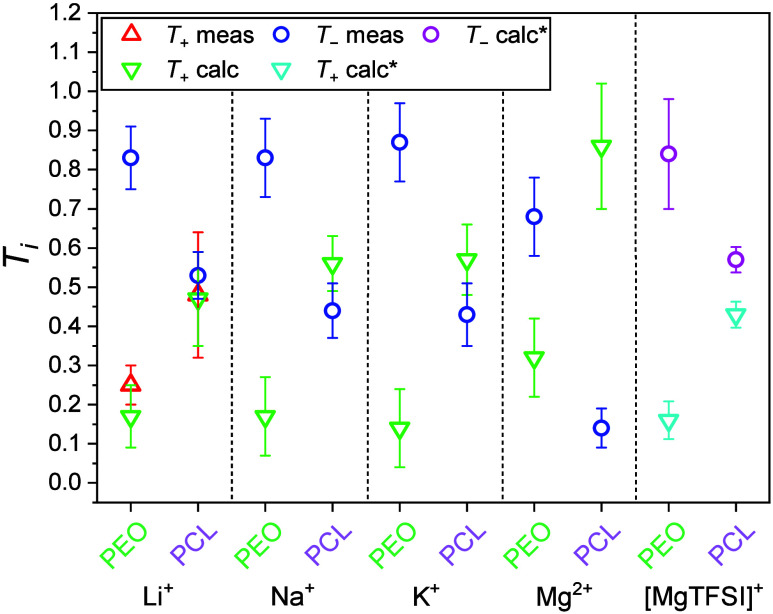
Calculated *T*_*i*_ for NaTFSI, KTFSI, and Mg(TFSI)_2_ in PEO and PCL determined by the combined eNMR/EIS method. LiTFSI system serves as a validation of the method. Measured *T*_Li_ are reused from ref (20) and *T*_*i*_ for the PEO systems are reused from ref (24). Numerical values of μ and *T*_*i*_ can be seen in Tables S2 and S3. The values in the rightmost column are calculated values obtained when the Mg system is treated as a monovalent system. For details of this treatment, see eq 10–13 and the associated discussion.

In Figure 4, the measured σ_tot_ along with the partial conductivities σ_–_ and σ_+_ (calculated from μ_*–*_, listed in Table S2) are shown. For the Li salt-containing electrolyte we compare the calculated cation conductivity (see green data point) to that resulting from a direct ^7^Li experiment (red data point) and find very good agreement, validating the approach. As seen, all conductivities for all monovalent ions are rather similar or at least within the same order of magnitude. For the divalent Mg, on the contrary, σ_tot_ is decreased, most probably due to strong interactions with other species. However, when determining the partial conductivities, it can be seen that the reduction of σ_–_ is more pronounced than that of σ_+_ in the Mg electrolyte, as compared to the monovalent cations. From the partial conductivities of the cation and anion, respectively, the resulting *T*_*i*_ of NaTFSI, KTFSI (both with *r* = [salt]/[monomer] = 0.1) and Mg(TFSI)_2_ (*r* = 0.05) in PCL are shown in Figure 5. Furthermore, *T*_*i*_ of the LiTFSI systems with *r* = 0.1 (from ref (20)), measured via ^7^Li eNMR measurements, are shown alongside the *T*_*i*_ derived according to the eNMR/EIS approach. The measured *T*_Li_ in Figure 5 is in accordance with the value that was determined by the eNMR/EIS approach, which serves to validate the reliability of the method. Figure 3 and 4 also include the recently published values of the PEO-based systems with NaTFSI, KTFSI and Mg(TFSI)_2_, with the same *r* as for the PCL systems, for the purpose of a comparison of the systems.^24^ Note that in addition Figure 3–5 include values for the Mg–TFSI pair, which are calculated under the assumption that Mg(TFSI)_2_ is only partly dissociated into [MgTFSI]^+^ and TFSI^–^. This scenario is discussed further below.

As previously demonstrated for Li-based systems,^20^ we note a clear correlation between *T*_+_ and the coordination strength of the polymer for NaTFSI, KTFSI or MgTFSI_2_. For all salts, we see a higher Δ*G*^0^ in PEO compared to PCL, indicating a stronger ion coordination strength in PEO, concomitant with a lower *T*_+_ compared to PCL and vice versa. When PCL is used as the polymer host, an increase in the *T*_+_ is seen for all three post-Li salts, in analogy to the previous finding for Li. Furthermore, comparing the different cations, similar *T*_+_ values around 0.5–0.6 are obtained for Na and K in PCL, within the margin of error for the value seen for Li.

In contrast, the *T*_+_ for Mg in PCL is unexpectedly high with a value of 0.86. Compared to Li^+^, Mg^2+^ has approximately twice the charge density, and could therefore be expected to interact much more strongly with the polymer host, thereby leading to a far stronger immobilization and lower transference. Even in the strongly coordinating PEO, the *T*_+_ for Mg is seen to increase compared to that of Li. This can most reasonably be explained by considering Mg mainly being present and coordinated by the polymer not in the form of Mg^2+^ but in the form of monovalent [MgTFSI]^+^ pairs.^24^ When detecting the electrophoretic mobility, the resulting value represents a fast exchange average value of all species containing the constituent TFSI. Therefore, the strikingly low magnitude of μ_–_ (far lower than that of the same anion TFSI in all other samples) (Figure 3), and consequently low σ_–_ in Figure 4, is likely the consequence of averaging the mobility of the anionic TFSI species with that of [MgTFSI]^+^ pairs. While free TFSI anions migrate toward the positive electrode, the net positively charged [MgTFSI]^+^ pairs migrate toward the negative electrode, resulting in a very low average value of μ_*–*_, which ultimately renders an inflated *T*_+_.

**Figure 6:**
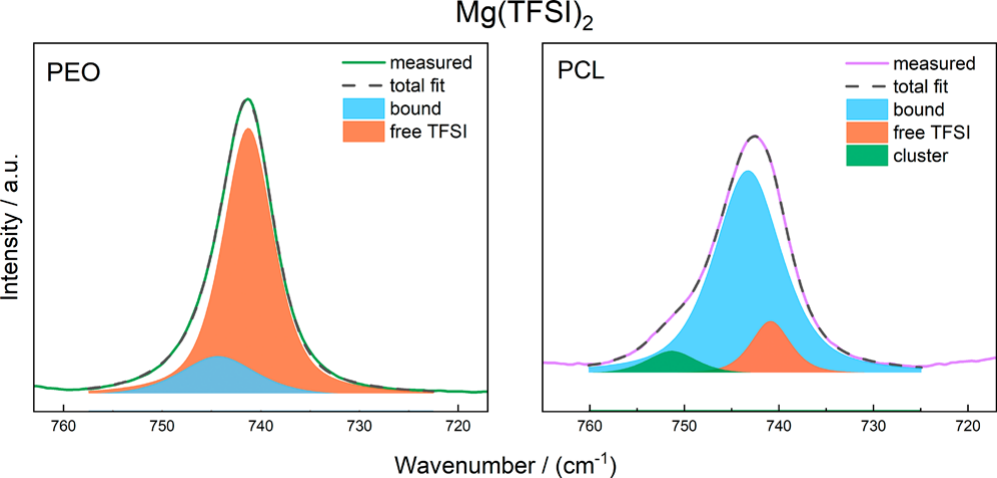
Raman spectra of Mg(TFSI)_2_ in PEO (left) and PCL (right), and their deconvolution into free anions, bound anions, and larger clusters.

The structural picture of Mg as [MgTFSI]^+^ cationic pairs is supported by Raman measurements, where free and coordinated anions can be distinguished. In the case of PEO indeed the fraction of bound TFSI and thus ion pairs is enhanced for Mg as compared to the electrolytes with monovalent cations (Figure 6 and S4, Table S4). Along the same lines, the occurrence of even larger cluster species is observed for Mg in the case of PCL (Figure 6 and S5, Table S5). More evidence supporting the presence of clusters can also be found in the literature.^15,28,29^ However, it is important to note that the picture of coordination seen in Raman spectroscopy is very different from the picture seen in eNMR, as both methods relate to very different time scales. Raman spectroscopy detects species regardless of whether they contribute to ion transport or not. For example, a large fraction of free ions detected by Raman does not mean that ion transport will be dominated by such unclustered ions. Thus, the speciation from Raman will be a poor representation of the overall dynamics of the system. In the present case, however, both structural and dynamic speciation highlights a significant role of [MgTFSI]^+^ cationic pairs.

Considering the high charge density of Mg^2+^, it makes sense to expect its ionic mobility to be less than that of Li^+^ in both polymer matrices, and several authors have suggested that Mg^2+^ is essentially immobile in PEO.^30^ In contrast, the mobility of Mg obtained via the eNMR/EIS approach, assuming full dissociation, exceeds that of all other cations, especially in the case of PCL (Figure 3). This poses an additional argument for the treatment of the Mg salt as not fully dissociated. Instead, assuming a monovalent model consisting of [MgTFSI]^+^ pairs and free TFSI counteranions, a calculation of *T*_+_ of the [MgTFSI]^+^ cation can be performed. In this model, the net TFSI mobility, which was measured by probing ^19^F with eNMR, is assumed to be an average of the contributions from free TFSI anions and TFSI in cationic Mg–TFSI pairs. Since the concentrations of the positive and negative species in such a monovalent system are equal, the measured μ_–_^net^ can be calculated as
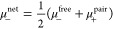
10By combining eq 6, 7 and 10, the total conductivity can be described in terms of the TFSI mobilities (μ_–_^free^ and μ_+_^pair^) in the system:
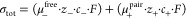
11Here, *c*_*–*_ and *c*_+_ will both be equal to the Mg concentration, and by taking the charge number (±1) into account, we get
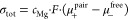
12By combining eq 10 with eq 12, the mobility of the pairs–in other words, the mobility of Mg–can be derived from μ_–_^net^ and σ_tot_:
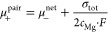
13Finally, an alternative *T*_+_ can be calculated with eq 6–9 for Mg assuming the formation of partly dissociated Mg–TFSI pairs. For both polymers, we arrive at a decreased transference number of 0.43 for PCL and 0.16 for PEO (Figure 5), respectively, clearly within the same range as found for the other (monovalent) salts in the same polymers.

Based on the available data, a direct conclusion on the validity of either dissociation model is not straightforward. While the Raman spectra show notable ion pairing and clustering for Mg(TFSI)_2_ when compared to the other salts, the Mg–TFSI association is more pronounced in PCL than in PEO, in line with the weaker coordination in PCL. On the other hand, as already noted, there is likely a large difference in mobility between the Mg species Mg^2+^ and [MgTFSI]^+^ because of an expected strong association between the small, divalent Mg^2+^ and the coordinating polymers. However, the static picture given by the Raman data does not necessarily correlate with the dynamics observed with eNMR. Nevertheless, even the transport behavior shows an enhanced ion pairing in the PCL system for all cations as supported by the effective charge of TFSI (ε_TFSI_) of ∼0.5 (Figure S6). The latter was calculated via the ratio μ^eNMR^/μ^pfg-NMR^ from diffusion coefficients (Figure S7) and apparent mobilities (Table S6), and it is far lower as compared to ∼0.9 in the PEO system,^24^ implying a smaller amount of free TFSI in the PCL system. Caution should, however, be exercised in the interpretation of these values in the case of Mg, since the above discussion suggests that the Mg systems do not simply behave as Mg^2+^ + 2 [TFSI]^−^, and therefore ε_TFSI_ does not directly scale with the degree of ion dissociation.

## Conclusions

These investigations confirm the applicability of the eNMR/EIS method not only for “beyond-Li” cations, but also for diverse polymer hosts with different coordinating characteristics. Furthermore, the correlation between *T*_+_ and the coordination strength has been verified for this wide range of systems, for which this correlation has only been hypothesized in previous studies due to the unavailability of methods to reliably determine *T*_+_ experimentally for beyond-Li cations. These measurements provide interesting insights into the discussion of the speciation of the Mg salt, by showing that charge transport by Mg-containing species is not negligible. An (at least partial) speciation into [MgTFSI]^+^, while coordination to the polymer persists, appears the most likely scenario.

## Supporting Information Available

Additional FTIR and Raman spectra. Densities of samples. van’t Hoff plots and additional thermodynamic data. Numeric data on ion mobilities and transference numbers. Effective charges and diffusion coefficients (PDF)
