# Supplementary material for: Transference Numbers and Ion Coordination Strength for Mg2+, Na+, and K+ in Solid Polymer Electrolytes
Source: J Phys Chem C Nanomater Interfaces. 2024 Sep 23;128(39):16393–9. doi: 10.1021/acs.jpcc.4c04632 (PMC11457214; doi:10.1021/acs.jpcc.4c04632)
Supplement: Supplementary file 1 — jp4c04632_si_001.pdf [file jp4c04632_si_001.pdf]

Supporting Information for

Transference Numbers and Ion Coordination Strength for Mg^{2+}, Na^+ and K^+ in Solid Polymer Electrolytes

Rassmus Andersson[1†], Caroline Mönich[2†], Guiomar Hernández[1], Monika Schönhoff[2] and Jonas Mindemark[1*]

[1]*Department of Chemistry – Ångström Laboratory, Uppsala University, Box 538, SE-751 21 Uppsala, Sweden*

[2]*Institute of Physical Chemistry, University of Münster, Corrensstraße 28/30, 48149 Münster, Germany*

[†] Equal contributions

* Corresponding author: jonas.mindemark@kemi.uu.se

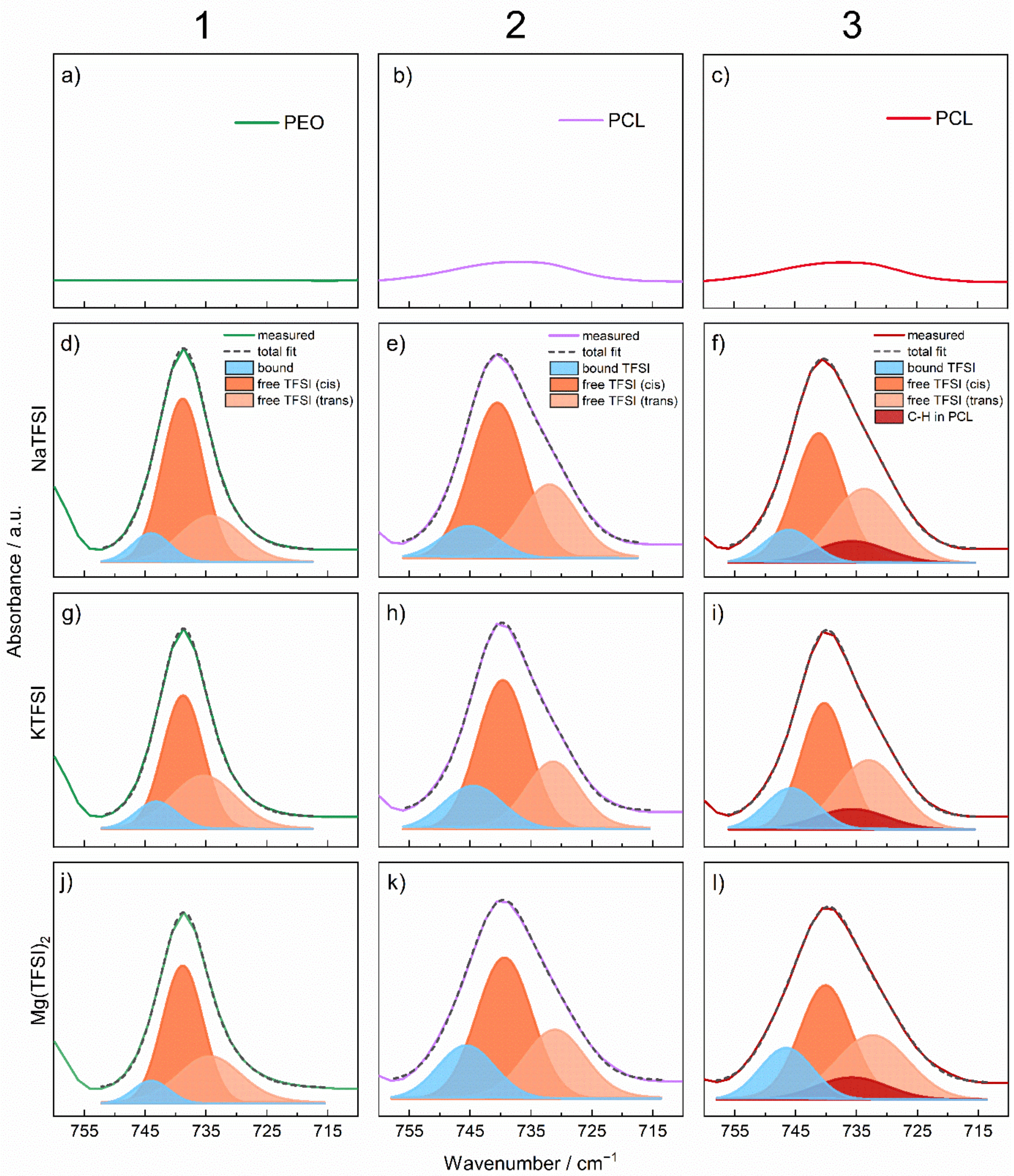

Figure S1. Deconvolution examples of the S-N-S vibration at 740 cm^{-1} of the TFSI anion in FTIR spectra. In column 1, the deconvolution of the PEO samples is shown; in columns 2 and 3, the deconvolution of the PCL samples is shown. The difference between columns 2 and 3 is that a peak vibration assigned to the C-H bending vibration in PCL is included in column 3. This peak is normalized to the total peak area for the S-N-S vibration. These representative examples for each sample are taken from triplicates at 85 °C.

Table S1. Densities (ρ) and concentrations of the respective cation c_+ and anion c_- in NaTFSI, KTFSI with r=0.1 or Mg(TFSI)$_2$ with r=0.05 in PCL (4000 g mol^{-1}) at 90 °C. * The lithium system serves as a comparison with data taken from (Ref. [1]).

	ρ / (g cm^{-3})	c_+ /mol L^{-1}	c_- / mol L^{-1}
**PCL + LiTFSI (*r*=0.1)**	1.13*	0.79*	0.79*
**PCL + Mg(TFSI)$_2$ (*r*=0.05)**	0.979 ± 0.001	0.34	0.68
**PCL + NaTFSI (*r*=0.1)**	1.08 ± 0.01	0.75	0.75
**PCL + KTFSI (*r*=0.1)**	1.07 ± 0.01	0.73	0.73

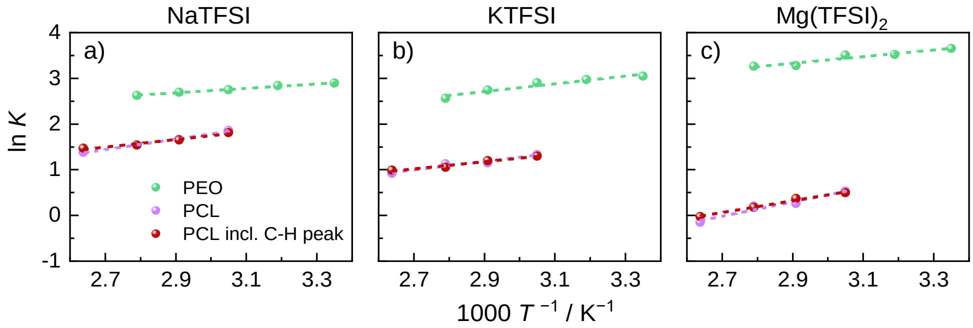

Figure S2. Van't Hoff plot examples of one measurement out of triplicates in the temperature range 25–85 °C for PEO and 55–100 °C for PCL. The linearity of the plot confirms that the enthalpy and entropy can be considered to be temperature-independent and can be extracted from the plot, both quantities are used to calculate the Gibbs free energy.

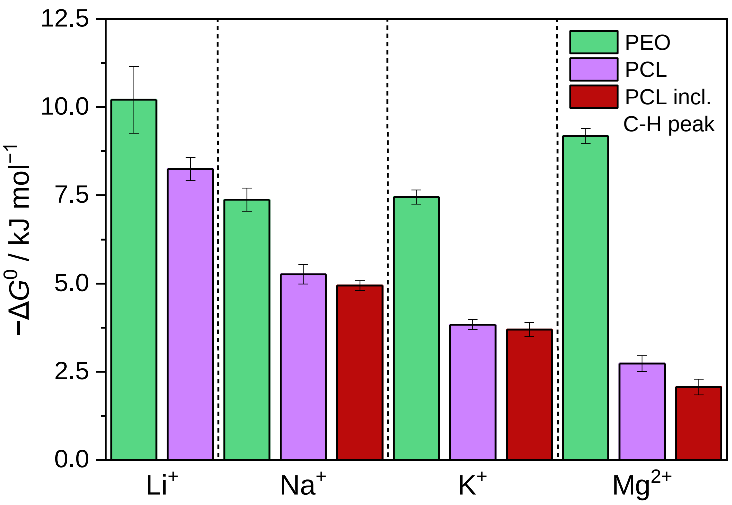

Figure S3. Calculated dissociation energies (ΔG^0) for Li^+, Na^+, K^+ and Mg^{2+} in PEO and PCL at 25 °C. The difference between the two PCL systems is that the red bars include a C-H bending vibration peak from PCL in the deconvolution (Figure S2).

Table S2. Mobilities of the anion (via ^{19}F eNMR) and the cation (calculated via the eNMR/EIS approach) of NaTFSI, KTFSI with r=0.1 or $Mg(TFSI)_2$ with r=0.05 in PCL or PEO (4000 g mol^{-1}). Calculated μ_- and μ_+ marked with * are numerical values obtained when the Mg system is treated as a monovalent system. Values for the PEO systems are reused from Ref. [2], whereas the values for the lithium systems marked with [¤] are taken from Ref. [1].

	**μ_+ meas / (10^{-10} m² V^{-1} s^{-1})**	**μ_- meas / (10^{-10} m² V^{-1} s^{-1})**	**μ_+ calc / (10^{-10} m² V^{-1} s^{-1})**	**μ_- calc* / (10^{-10} m² V^{-1} s^{-1})**	**μ_+ calc* / (10^{-10} m² V^{-1} s^{-1})**
**PCL + LiTFSI (*r*=0.1)**	1.51 ± 0.5 [¤]	−1.62 ± 0.2 [¤]	1.46 ± 0.3		
**PCL + NaTFSI (*r*=0.1)**		−1.51 ± 0.2	2.00 ± 0.2		
**PCL + KTFSI (*r*=0.1)**		−1.79 ± 0.3	2.40 ± 0.4		
**PCL + Mg(TFSI)$_2$ (*r*=0.05)**		−0.48 ± 0.16	3.01 ± 0.5	−3.97 ± 0.36	3.01 ± 0.16
**PEO + LiTFSI (*r*=0.1)**	1.80 ± 0.2	−5.50 ± 0.5	1.13 ± 0.6		
**PEO + NaTFSI (*r*=0.1)**		−5.58 ± 0.6	1.1 ± 0.6		
**PEO + KTFSI (*r*=0.1)**		−5.55 ± 0.5	0.93 ± 0.7		
**PEO + Mg(TFSI)$_2$ (*r*=0.05)**		−3.00 ± 0.4	1.45 ± 0.4	−7.45 ± 0.89	1.45 ± 0.40

Table S3. Numeric transference numbers of the anion T_- and cation T_+ calculated via the eNMR/EIS approach for NaTFSI, KTFSI with r = 0.1 or $Mg(TFSI)_2$ with r = 0.05 in PCL and PEO (4000 g mol^{-1}). Calculated T_- and T_+ marked with * are numerical values obtained when the Mg system is treated as a monovalent system. Values for the PEO systems are reused from Ref. [2], whereas the values for the lithium systems marked with [¤] are taken from Ref. [1].

	**T_+ meas**	**T_- meas**	**T_+ calc**	**T_- calc***	**T_+ calc***
**PCL + LiTFSI (*r*=0.1)**	0.48 ± 0.15 [¤]	0.53 ± 0.05 [¤]	0.47 ± 0.12		
**PCL + NaTFSI (*r*=0.1)**		0.44 ± 0.07	0.56 ± 0.08		
**PCL + KTFSI (*r*=0.1)**		0.43 ± 0.08	0.57 ± 0.09		
**PCL + Mg(TFSI)$_2$ (*r*=0.05)**		0.14 ± 0.05	0.86 ± 0.18	0.57 ± 0.03	0.43 ± 0.18
**PEO + LiTFSI (*r*=0.1)**	0.25 ± 0.05	0.83 ± 0.10	0.17 ± 0.10		
**PEO + NaTFSI (*r*=0.1)**		0.83 ± 0.10	0.17 ± 0.10		
**PEO + KTFSI (*r*=0.1)**		0.86 ± 0.10	0.14 ± 0.10		
**PEO + Mg(TFSI)$_2$ (*r*=0.05)**		0.68 ± 0.10	0.33 ± 0.10	0.84 ± 0.14	0.16 ± 0.10

Table S4. Deconvoluted peak ratios and positions of bound and free TFSI respectively for LiTFSI, NaTFSI, KTFSI and $Mg(TFSI)_2$ in PEO, derived from the Raman spectra in Figure S4.

	**PEO + LiTFSI**		**PEO + Mg(TFSI)$_2$**		**PEO + NaTFSI**		**PEO + KTFSI**	
	position / cm^{-1}	ratio / %	position / cm^{-1}	ratio / %	position / cm^{-1}	ratio / %	position / cm^{-1}	ratio / %
bound TFSI	745	11	744	19	745	11	744	14
free TFSI	741	89	741	81	741	89	741	86

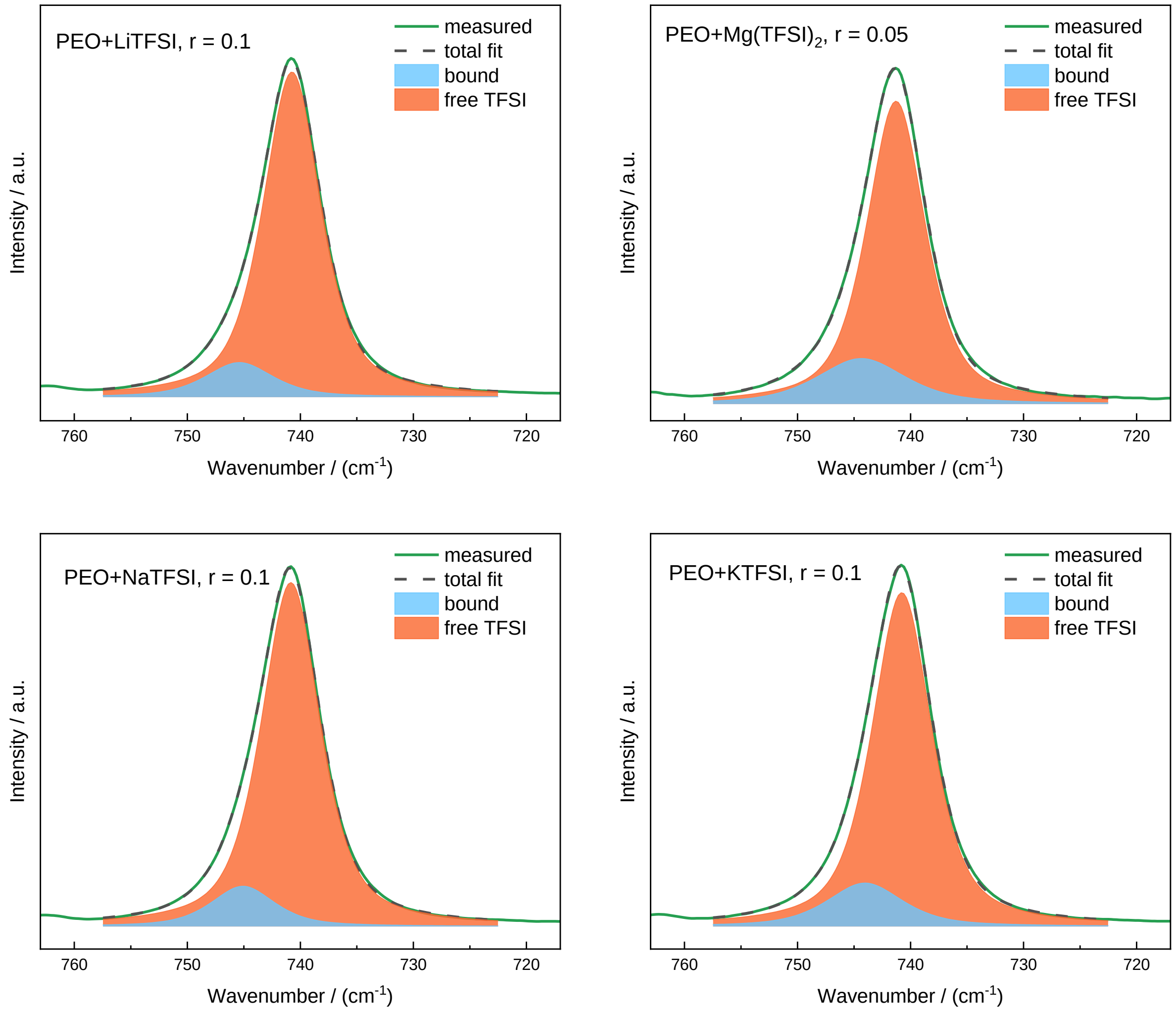

Figure S4. Raman spectra of LiTFSI, NaTFSI, KTFSI and $Mg(TFSI)_2$ in PEO, and their deconvolution into free anions and bound anions.

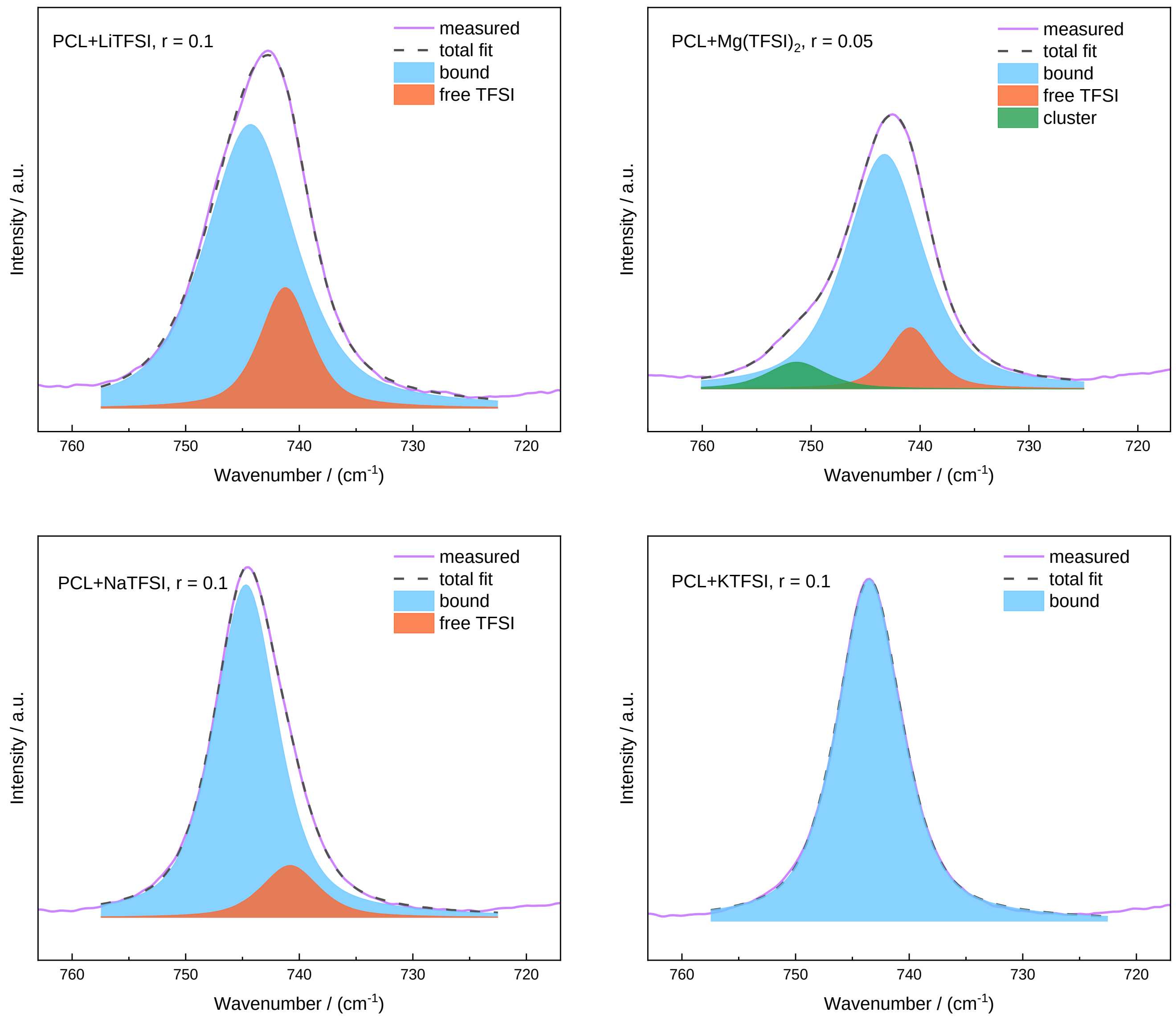

Figure S5. Raman spectra of LiTFSI, NaTFSI, KTFSI and $Mg(TFSI)_2$ in PCL, and their deconvolution into free anions, bound anions and larger clusters.

Table S5. Deconvoluted peak ratios and positions of bound, free and clustered TFSI, respectively, for LiTFSI, NaTFSI, KTFSI and $Mg(TFSI)_2$ in PCL, derived from the Raman spectra in Figure S5.

	**PCL + LiTFSI**		**PCL + $Mg(TFSI)_2$**		**PCL + NaTFSI**		**PCL + KTFSI**	
	position / cm^{-1}	ratio / %	position / cm^{-1}	ratio / %	position / cm^{-1}	ratio / %	position / cm^{-1}	ratio / %
bound TFSI	744	81	743	81	745	88	744	100
free TFSI	741	19	741	12	741	12	--	--
cluster	--	--	751	7	--	--	--	--

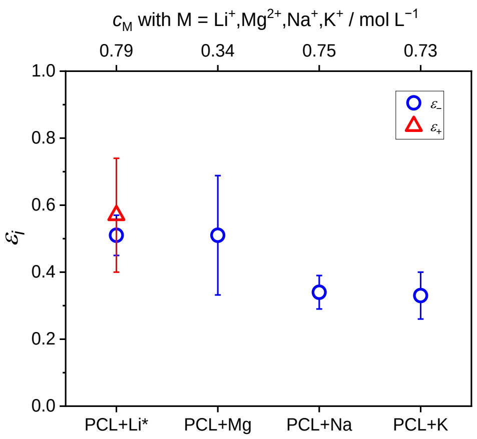

Figure S6. Effective charges (ε_i) of the anion calculated from the apparent mobilities (Table S6), via the diffusion coefficients by PFG NMR (Figure S8) and the electrophoretic mobility (via ^{19}F eNMR) of NaTFSI, KTFSI with $r = 0.1$ or $Mg(TFSI)_2$ with $r = 0.05$ in PCL (4000 g mol^{-1}) at 90 °C. The lithium system (*) serves as a comparison with data taken from Ref. [1].

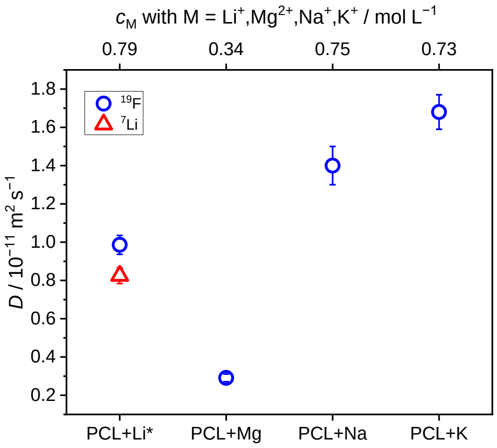

Figure S7. Diffusion coefficients of TFSI in NaTFSI, KTFSI with $r = 0.1$ or $Mg(TFSI)_2$ with $r = 0.05$ in PCL (4000 g mol^{-1}) at 90 °C. The diffusion coefficients were calculated from the apparent mobilities in Table S6. The lithium system (*) serves as a comparison with data taken from Ref. [1].

Table S6. Apparent mobilities determined by PFG-NMR ($\mu^{\mathrm{PFG\text{-}NMR}}$) of the anion in NaTFSI, KTFSI with *r*=0.1 or $Mg(TFSI)_2$ with *r*=0.05 in PCL (4000 g mol^{-1}) at 90 °C. The values for the lithium system serve as a comparison and are taken from (Ref. [1]).

The mobilities were calculated according to

$$\mu^{PFG-NMR} = \frac{z_{-}D_{-}}{k_{\mathrm{B}}T}$$

	$\mu_{-}^{PFG\text{-}NMR}$ / (10^{-10} m^2 V^{-1} s^{-1})	$\mu_{+}^{PFG\text{-}NMR}$ / (10^{-10} m^2 V^{-1} s^{-1})
**PCL + LiTFSI (*r*=0.1)**	−3.15 ± 0.2	2.64 ± 0.1
**PCL + $Mg(TFSI)_2$ (*r*=0.05)**	−0.93 ± 0.07	
**PCL + NaTFSI (*r*=0.1)**	−4.47 ± 0.3	
**PCL + KTFSI (*r*=0.1)**	−5.38 ± 0.3	

References

(1) Rosenwinkel, M. P.; Andersson, R.; Mindemark, J.; Schönhoff, M., Coordination Effects in Polymer Electrolytes: Fast Li^+ Transport by Weak Ion Binding. *J. Phys. Chem. C* **2020,** *124,* 23588–23596.

(2) Mönich, C.; Andersson, R.; Hernandez, G.; Mindemark, J.; Schönhoff, M., Seeing the Unseen: Mg^{2+}, Na^+, and K^+ Transference Numbers in Post-Li Battery Electrolytes by Electrophoretic Nuclear Magnetic Resonance. *J. Am. Chem. Soc.* **2024,** *146,* 11105–11114.
